# Supplementary material for: Towards Reference Values for Malondialdehyde on Exhaled Breath Condensate: A Systematic Literature Review and Meta-Analysis
Source: Toxics. 2022 May 18;10(5):258. doi: 10.3390/toxics10050258 (PMC9147097; doi:10.3390/toxics10050258)
Supplement: Supplementary file 1 [file toxics-10-00258-s001.zip › toxics-1682604-supplementary.pdf]

Author, year, country	Study objectives	Population studied and number of participants	Control population	Method of EBC collection and analysis	Main findings	Quality score
**Aksu, 2012, Turkey [1]**	to investigate lower airway inflammation using malondialdehyde and total protein measurement in exhaled breath condensate	12 mild asthma, 53 persistent rhinitis, and 16 concomitant asthma and rhinitis	13 controls, 5 males and 8 women	EBC was collected with a homemade apparatus for max 15 mins at 10.00 am, no nose clip, immediately stored at -80°C	MDA and total protein levels in EBC did not differ between the groups	14 moderate
**Andreoli, 2003, Italy [2]**	method developement	none	2 nonsmoking controls and 13 asymptomatic smokers	EBC collected on EcoScreen, 15 mins with nose clip and then quickly stored at -80°C	MDA concentrations were consistent with those determined in EBC using an independent HPLC FLD method, mass spectrometry to EBC analysis could play a fundamental role in the validation of EBC as a new accessible biological matrix and in the definition of standardized EBC collection	12 moderate
**Antus, 2015, Hungary [3]**	to investigate oxidative stress in cystic fibrosis (CF) patients	40 patients with CF	25 controls, 13 males and 12 females	EBC collection with an EcoScreen condenser using MDA reagent kit, MDA concentrations in EBC were measured with an isocratic HPLC system	MDA concentration in EBC, sputum and plasma did not correlate; there also no correlation between MDA levels in these matrices and FENO and lung function	19 high

**Antus, 2013, Hungary [4]**	to investigate MDA levels in patients with acute COPD exacerbation	55 patients with COPD	20 healthy controls, ex-smokers, 10 women and 10 males	EBC collected for 10 min EcoScreen condenser ,samples were stored frozen at -80°C before analysis, isocratic HPLC system using MDA reagent kit	No correlations between EBC, sputum and plasma MDA and FENO, lung functions and blood gas parameters either in stable or acute disease	12 moderate
**Antczak, 1997, Poland [5]**	to determine wether asthmatic patients exhale more H2O2 and TBARS than healthy subjects	21 asthmatic patients	10 healthy subjects, 6 males and 4 females	EBC collected in a tube installed, in a polystyrene foam container filled with ice and salt, for 20 mins and aliquots were transferred in Eppendorf tubes stored at -80°C for not longer than 7 days until measurement. TBAR species in expired breath condensate was determined according to the method of YAGI	H2O2 was 26 times higher in asthmatic EBC, as it was for the TBARS, these findings were statistically significant	15 moderate
**Araneda, 2005, Chile [6]**	to investigate lung oxidative stress in subjects exercising in moderate and high altitude	none	6 healthy males	EBC collected with self-constructed device for 20 mins, condensated ad -5°C, and stored in liquid nitrogen. MDA was determined with HPLC according to Larstad	Lung OS may constitute a pathogenic factor in acute mountain sickness	11 moderate

**Araneda, 2012, Chile [7]**	to investigate the impact of an endurance race on pulmonary pro-oxidative formation and lipoperoxidation	41 healthy recreational runners divided in 3 groups according to the races they decided to run (10, 21.1 and 41.2 km) non smokers	two groups (10 and 42.2 km) with MDA in EBC pre-race Data was available just for these subjects	EBC collected with self-constructed device for 10 to 20 mins, with nose clip, condensated ad -5°C, and stored in liquid nitrogen. MDA was determined with HPLC according to Larstad	Intense prolonged exercise favors the increase in pulmonary pro-oxidative levels, with no modifications on lipoperoxidation. Running time relates to the magnitude of acute post exercise pro-oxidative formation	12 moderate
**Araneda, 2013, Chile [8]**	to investigate pro-oxidative, lipid peroxidation and inflammation in EBC,in subjects running 10 km	none	10 physically active healthy subjects, 9 males and 1 female; 20 mins before the race	EBC collected with self-constructed device for 10 to 15 mins, with nose clip, condensated ad -5°C, and stored in liquid nitrogen. MDA was determined with HPLC according to Larstad	unlike the previous results obtained in amateur runners, in physically active subjects, 10 km produces an increase in oxygen- and nitrogen-derived pro-oxidative species, without early per-oxidation	7 low
**Barregard, 2007, Sweden [9]**	To examine whether short-term exposure to wood smoke in healthy subjects affects markers of pulmonary inflammation and oxidative stress.		13 healty subjects, 6 males and 7 women, EBC measured prior to exposure	EBC was collected with an Rtube, with nose clip, until 80 liters were collected, samples were frozen at -20°C. MDA was determined with HPLC according to Larstad	wood smoke exposure affects the respiratory tract, especially the lower airways	10 moderate
**Bartoli, 2011, Italy [10]**	to assess usefulness of MDA in EBC in different groups of pulmonary diseases	64 subjects with asthma, 19 with bronchiectasies, 73 with COPD, 38 with idiopathic pulmonary fibrosis	14 healthy nonsmoking subjects	EBC was collected on an EcoScreen device, , during 15 mins, samples were immediately at - 80°C and analyzed within 6 months, with HPLC according to Larstad	Subjects with chronic airway disorders have increased levels of MDA in EBC; that MDA concentrations in EBC are related to FEV1 and neutrophilic inflammation, particularly in COPD patients.	15 moderate

**Brand, 2013, Germany [11]**	to investigate if short-term exposure to welding fumes results in changes in lung function and early stages of inflammatory reactions.		12 healthy non smoking males	EBC was collected on ECOSCREEN wearing a nose clip, for 20 mins, samples stored at -80°C until analysis. MDA was derivatized with DNPH and then separated by HPLC and determined by mass spectrometry	In healthy, young subjects neither changes in spirometry nor changes in inflammatory markers measured in exhaled breath condensate could be detected after short term exposure	12 moderate
**Caglieri, 2006, Italy [12]**	to investigate Chromium levels in EBC of workers exposed to Cr(VI) and to assess their relationship with biochemical changes in the airways by analyzing EBC biomarkers of oxidative stress	24 chrome-plating workers	25 controls, 13 males and 12 females; 5 ex-smokers and 20 non smokers	EBC was collected on TURBO DECCS, condensation temperature of -5°C, during 15 mins; MDA-EBC was measured by tandem liquid chromatography–mass spectrometry	Cr-EBC levels correlated with those of H2O2-EBC and MDA-EBC, as well as with urinary Cr levels.	14 moderate
**Casimirri, 2015, Italy [13]**	To evaluate if exposure to cleaning agents causes OS and to explore EBC biomarkers linked to demographic characteristics	40 cleaning agents in the same hospital as the controls; 12 currents smokers and 5 ex-smokers	40 controls, 10 smokers, 22 non-smokers and 8 ex-smokers, hospital administrative personnel	EBC was collected on TURBO DECCS, condensation temperature of -5°C, during 15 mins and then stored at -80°C until analysis	Professional exposure to chlorinated agents increases EBC biomarkers of oxidative stress and inflammation	15 moderate

**Corradi, 2003, Italy [14]**	to evaluate if aldehydes could be measured in EBC, to assess the influence of sampling procedures, to compare levels of sifferent pulmonary disease groups with those of control group	20 patients with stable COPD 18 men and 2 women	12 smoking controls (9 male and 3 women) and 20 non-smoking (17 men and 3 women)	EBC was collected on a Tygon Tube immersed in thawing ice, and then frozen to -80°C, no nose clip, MDA measured with LC-MS tandem	aldehydes were identified in EBC, all, but nonal were lower in control groups	16 moderate
**Corradi, 2002, Italy [15]**	to investigate short-term exposure to ozone (O3) induces changes in biomarkers of lung inflammation and oxidative stress in EBC		22 non smoking healthy controls (12 male and female)	EBC was collected on ECO SCREEN, during 15 mins, frozen at - 80°C, MDA was measured as TBARS according to Nowak	a single 2-h exposure to 0.1 ppm of O3 induces changes in biomarkers of inflammation and oxidative stress in those susceptible	11 moderate
**Corradi, 2004, Italy [16]**	to compare aldehyde levels resulting from lipid peroxidation in EBC and induced sputum (IS) supernatant of subjects with asthma and chronic obstructive pulmonary disease	21 subjects with COPD, 10 asthmatics	9 healthy non-smoking controls; 8 women and 1 male	EBC was collected on a two glass chamber device from Incofar; no nose-clip for 20 mins, samples were stored at -80°C and MDA measured according to Larstad	aldehydes can be detected in both exhaled breath condensate and supernatant of induced sputum, but that their relative concentrations are different and not correlated with each other	12 moderate

**Doruk, 2011, Turkey [17]**	to investigate oxidative stress in the lungs associated with tobacco smoke and to evaluate the effect of this stress with pulmonary function tests		69 healthy subjects divided in 3 groups according to their exposure to tobacco smoke: 26 current smokers (23 male, 3 female), 21 subjects (15 male, 6 female) who did not smoke within the last year but had second-hand smoking and 22 (100 male and 12 female) with no tobacco smoke exposure	EBC on ECOSCREEN for 15 mins,wearing a noseclip, samples stored at -70°C; MDA measured as TBARS	The levels of MDA, 8-OHdG, SOD and GSH-Pxwere higher in smokers. NO levels gradually increased fromGroup I to Group III. MDA levels were lower in Group IIIthan Group II	14 moderate
**Goldoni, 2004, Italy [18]**	to investigate whether EBC can be used as a suitable matrix to assess target tissue dose and effects of inhaled cobalt and tungsten, using EBC malondialdehyde (MDA) as a biomarker of	33 workers exposed to Co and W in workshops producing either diamond tools or hard-metal mechanical parts	16 controls (11 male and 5 females)	EBC was collected on a homemade apparatus, during 10 mins, samples were transported in ice to lab and then stored at -80°C; MDA was measured by liquid chromatography tandem mass spectrometry (LC-MS/MS)	MDA levels were increased depending on cobalt concentration and were enhanced by coexposure to tungsten.	11 moderate

	pulmonary oxidative stress.					
**Goldoni, 2013, Italy [19]**	to compare the concentration of several biomarkers in whole (W-EBC) and fractionated EBC (A-EBC)		45 healthy subjects (10 males and 35 females); 6 smokers and 39 non-smokers	EBC collected on a homemade apparatus for the fractioned EBC and a TURBODECCS for the whole EBC for 15 mins without a noseclip, EBC was centrifuged and then stored at -80°C; MDA was measured by liquid chromatography tandem–mass spectrometry	H2O2 , 8-isoprostane, malondialdehyde and 4-hydroxy-2-nonhenal were all higher in W-EBC, suggesting a contribution from the upper airways to oxidative stress biomarkers in apparently healthy subjects.	10 moderate
**Goldoni, 2005, Italy [20]**	test the effect of condensation temperature on the parameters of exhaled breath condensate and the levels of selected biomarkers.		24 healthy subjects (13 males and 11 females)- 3 ex-smokers and 21 non-smokers	EBC was collected on a TURBODECCS, during 10 mins, at different temperatures, samples were sentrifuged and then stored at -80°C, MDA was measured by liquid chromatography-mass spectrometry tandem (LC-MS/MS) within 2 weeks	cooling temperature of exhaled breath condensate collection influenced selected biomarkers	13 moderate

**Gong, 2014, USA/China [21]**	to compare ultrafine particles (UFPs) and fine particles (PM2.5) in their associations with biomarkers reflecting multiple pathophysiological pathways linking exposure and cardiorespiratory events		125 healthy non-smoking individuals, initially 64 males and 64 women working on a campus near Peking's University Health Sciences Centre	EBC on ECOSCREEN , collected for 20 mins, with noseclip, aliquotes stored at -70°C, MDA was measured as MDA-TBA in HPLC	associations of certain biomarkers with UFPs had different lag patterns compared to those with PM2.5, suggesting that the ultrafine size fraction and the fine size fraction of PM2.5 are likely to affect PM-induced pathophysiological pathways independently.	15 moderate
**Graczyk, 2016, Switzerland [22]**	to investigate time course changes of particle-associated oxidative stress in exposed tungsten inert gas welders.		20 nonsmocking healthy volunteers, wth les than 1 year of apprentice in welding	EBC was collected on an Rtube, during 10 mins, while wearing a noseclip, EBC samples were stored at -70°C; MDA was measured by HPLC separation and fluorescence detection TBARS	A 60-min exposure to TIG welding fume in a controlled, well-ventilated setting induced acute oxidative stress at 3 h post exposure in healthy, non-smoking apprentice welders not chronically exposed to welding fumes.	10 moderate
**Gube, 2010, Germany [23]**	to investigate the effect of welding as well as the impact of smoking and protection measures on biological markers in EBC	45 male welders	24 healthy male non exposed	EBC was collected on ECOSCREEN, wearing a noseclip, as long as 200l oh exhaled breath was collected, then stored at -80°C; MDA was measued with a previously described but slightly changed method- MDA derivatized with DNPH and separated by HPLC,	welders showed signiWcantly increased concentrations of all these parameters at baseline compared to non-exposed controls	14 moderate

				then tandem-mass spectrometry		
**Inonu, 2012, Turkey [24]**	to evaluate the differences in the burden of oxidative stress in patients with COPD, smokers, and non-smokers by measuring hydrogen peroxide (H2O2), malondialdehyde (MDA), and 8-isoprostane levels in EBC	25 male COPD smokers	26 smokers and 29 non-smokers males healthy	EBC collected on EcoScreen, 15 mins with nose clip and then quickly stored at -70°C for 6 months, MDA was measured a commercial kit in fluorescence detector in HPLC	Even if respiratory function tests are within normal limits, oxidant burden in lungs of smokers is equivalent to that in COPD patients. 8-isoprostane could be useful in assessing symptom severity and health status of COPD patients	16 moderate

**Larstad, 2001, Sweden [25]**	to develop a method of MDA quantification	29 patients with asthma, 7 of which with wheezing	15 without asthma	EBC was collected on ECOSCREEN, during 4 mins with a nose clip, stored at -20°C until analysis, MDA was measured using TBS at 95°C and then separated by HPLC and MDA-TBA measured by fluorescence	no statistically significant difference between patients with asthma and patients without asthma. However, among females, subjects with asthma had higher MDA levels as compared to females without asthma. The use of the method when studying airway inflammation has to be further evaluated	12 moderate
**Laumbach, 2014, USA [26]**	to determine if exposure to traffic-related pollutants particles (TRAPs) during commuting causes acute oxidative stress in the respiratory tract or changes in heart rate variability		21 young volunteers, 15 males and 6 women	EBC collected with ECOSCREEN device, for 20 mins, samples were frozen to -80°C, MDA analysis,was performed by using a mixture of EBC, phosphoric acid, and thiobarbituric acid, was heated and injected into an HPLC-fluorescence system	Increases in markers of oxidative stress in EBC may represent early biological responses to widespread exposures to TRAPs particles that affect passengers in vehicles on heavily trafficked roadways	9 low
**Lee, 2015, Korea [27]**	to investigate the actual health effects of multi wall carbon nanotubes in manufacturing workers	9 male carbon nanotubes manufacturing workers, 5 smokers	4 office workers, 3 male, 1 female; 2 smokers	EBC was collected on an Rtube, during 10 mins, with a noseclip, stored in dry ice for 2 weeks. MDA was measured using fluorescence HPLC	analyzed biomarkers in the MWCNT manufacturing workers were significantly higher than those in the office workers; MDA, n-hexanal and molybdenum could be useful biomarkers of MWCNT exposure.	10 moderate

**Majewska, 2004, Poland [28]**	To determine whether concentrations of H2O2 and TBARs in EBC is elevated and correlate with systemic response to pneumonia during 10 days of hospital treatment	43 inpatients with community acquired pneumonia; 12 females/ 31 males	20 healthy non smoking controls, 6 females and 14 males	EBC was collected on a device from Jaeger, cooled with ethanol at -9°C, during 20 mins, wearing a nosclip, stored on ice until measurement was performed. MDA was measured as TBARS	Pneumonia is accompanied by oxidative stress in airways that moderately correlates with intensity of systemic inflammatory response. Determination of H2O2 in EBC may be helpful for non-invasive monitoring of oxidants production during lower respiratory tract infection	13 moderate
**Nowak, 2001, Poland [29]**	to investigate the concentration of H2O2 and TBARs in EBC and influecing factors		58 healthy volunteers, 18 smokers, 40 non smokers, 31 males and 27 women	EBC was collected on a device from Jaeger Toennies, during 20 mins, wearing a noseclipiately used. MDA was measured as TBARS spectrofotometrically	Neither moderate exercise nor one puff of salbutamol nor ipratropium influenced significantly the concentration of H2O2 and TBARs in EBC. Only 4 of 120 EBC specimens from never smoked subjects revealed detectable levels of TBARs. Cigarette smokers exhaled more TBARs	11 moderate

**Pelclova, 2018, Czech Republic [30]**	to investigate lung oxidative stress in workers handling nanocomposites	19 nanocomposite-synthesizing and processing researchers (14 men, 5 women, all non-smokers)	19, 13 men, 6 women, all non-smokers	EBC was collected on ECOSCREEN, until a minimum of 120 l of exhaled breath, wearing a noseclip, immediately stored at -80°C. MDA was measured	Significant associations were found between working in nanocomposite synthesis and EBC biomarkers. More research is needed to understand the contribution of nanoparticles from nanocomposite processing in inducing oxidative stress, relative to other co-exposures generated during welding, smelting, and secondary oxidation processes	12 moderate
**Pelclova,2014, Czech Republic [31]**	To search for optimal markers in the exhaled breath condensate (EBC), plasma and urine that would reflect the activity/ severity of occupational asthma (OA) after the withdrawal from the exposure to the allergen	43 subjects with previously diagnosed immunological OA 18 men, 25 women	20 subjects, working as office or health care employees and having no symptoms of asthma, 10 men, 10 women,	EBC was collected for 15–20 min with EcoScreen, wearing a noseclip, samples were stored at -80°C for max 2 months, MDA was measured with LC-ESI-MS/MS	improvement in OA is very slow and objective impairments persist years after removal from the exposure.Cysteinyl LTs and 8-ISO in EBC and 8-ISO in plasma might enrich the spectrum of useful objective tests for the follow-up of OA.	12 moderate
**Pelclova,2016, Czech Republic [32]**	to investigate the utility of oxidative stress biomarkers in EBC in iron oxide pigment	14 workers males, 43 ± 7 years, 43% smokers	14 males, 39 ± 4 years, 50% smokers, non-exposed	EBC was collected for 15 mins with EcoScreen, wearing a noseclip, samples were stored at -80°C, MDA was measured with LC-ESI-MS/MS	Almost all markers of lipid, nucleic acid and protein oxidation were elevated in the EBC of workers comparing with control subjects. Markers in urine were not elevated	10 moderate

	production workers					
**Ricelli, 2018, Italy [33]**	to investigate the Cromium and Nickel content of EBC of stainles stell (SS) tungsten inert gas (TIG) welders, and relate their concentrations with oxidative stress and inflammatory biomarkers.		100 SS welding workers aged 18–65 years; Smokers/non-smokers/ex-smokers 33/36/31 , values were considered pre-shift	EBC was collected on ECOSCREEN, during 15 mins, and stored at -80°C, MDA was determined by tandem LC-MS/MS	given the weak relationship between the biomarkers and effects of exposure, we speculate that other substances generated during SS TIG welding also play a role in generating lung oxidative stress.	11 moderate
**Rundell, 2008, USA [34]**	to investigate PM1 inhalation during exercise on lung function, exhaled nitric oxide (eNO), and total nitrate (NO3), S-nitrosoglutathione (GSNO), and malondialdehyde (MDA) in EBC		Twelve physically fit, nonasthmatic, nonsmoking males	EBC was collected on ECOSCREEN, during 15 mins, wearing a noseclip and stored at -80°C; MDA was measured after derivatization with TBA by HPLC fluorescence	MDA increased 40% after low PM exercise, high PM1 inhalation during exercise caused a reduced alveolar contribution to eNO; NO3 and eNO variables were decreased and were related to impaired lung function	9 low

**Sakhvidi, 2015, Iran [35]**	to measure exhaled breath malondialdehyde (EBC-MDA) in workers exposed to dust containing silica and at its comparison with the non-exposed control group.	25 male workers in a ceramic factory	25 male subjects from administrative departments of the same factory	EBC was collected with a home made apparatus, during 5 mins, wearing a noseclip, samples were stored in freezer at -20° until examination;MDA was analyzed by an HPLC equipped with a fluorescent detector	significant correlation between respirable dust exposure intensity and the level of EBC-MDA of the exposed subjects, but no significant correlation with lung functions	13 moderate
**Sauvain, 2014, Switzerland [36]**	to evaluate the feasibility of using exhaled breath condensate (EBC) from healthy volunteers for (1) assessing the lung deposited dose of combustion nanoparticles and (2) determining the resulting oxidative stress		15 healthy nonsmokers	EBC collected on a Rtube, during 10 mins, wearing a noseclip, a aliquote was stored at -78°C, MDA was derivatized with TBA then measure with HPLC fluorescence	results suggest two phases of oxidation markers in EBC: first, the initial deposition of particles and gases in the lung lining liquid, and later the start of oxidative stress with associated cell membrane damage	11 moderate
**Stockfelt, 2012, Sweden [37]**	to examine airway effects of two kinds of wood smoke in a chamber study		16 healthy never-smoking subjects (8 men and 8 women), 3 excluded for respiratory problems before the experiment	EBC collected according to Barregard; MDA measured always according to Barregard	relatively low levels of wood smoke exposure induce effects on airways	8 low

**Syslova, 2009, Czech Republic [38]**	to develope a sensitive method for a parallel, rapid and precise determination of the most prominent oxidative stress biomarkers for patients with silica or asbestos disease	20 subjects with previous exposure to silica or asbestos and related disease	10 subjects	EBC was collected on ECOSCREEN, for 5-10 mins, wearing a nose-clip, frozen at -80°C and stored max 1 month, MDA was measured with optimized LC–ESI-MS/MS	The difference in concentration levels of biomarkers between the two groups was perceptible in all the body fluids (the difference observed in an exhaled breath condensatewas statistically most significant).	12 moderate
**Szkudlarek, 2003, Poland [39]**	to test whether exhalation of H2O2 and TBARs by healthy subjects depends on reactive oxygen species generation from blood phagocytes		41 healthy, never smoked subjects (mean age 20.770.8 years,18 men, 23 w omen)	EBC was collected on a home made mouth piece connected to a glas tube cooler; wearing a noseclip, during 20 mins; MDA was measured as TBARS according to Nowak	No association between exhaled TBARs and blood phagocytes activity was found.	10 moderate
**Pelclova, 2020, Czech Republic [40]**	to analyze biomarkers in EBC of nanocomposites workers and understand abour health effects	20 researchers handling nanocomposites ; 15 men and 5 women, 19 non smokers, 41.8 mean age	21 controls; 15 men, 6 women, office emplyees; 19 non-smokers, 42.7 y.o	EBC was collected on ECOSCREEN and stored at -80°C, MDA was measured with LC-ESI-MS/MS	among inflammation markers, LT4 and tumor necrosis factor were the most useful	10 moderate

References

1. Aksu K, Kurt H, Gündüz E, Değirmenci I, Kurt E. Inflammatory markers in exhaled breath condensate in patients with asthma and rhinitis. Tuberk Toraks. 2012;60(4):321-6.

2. Andreoli R, Manini P, Corradi M, Mutti A, Niessen WMA. Determination of patterns of biologically relevant aldehydes in exhaled breath condensate of healthy subjects by liquid chromatography/atmospheric chemical ionization tandem mass spectrometry. Rapid Commun Mass Spectrom. 15 avr 2003;17(7):637-45.

3. Antus B, Drozdovszky O, Barta I, Kelemen K. Comparison of Airway and Systemic Malondialdehyde Levels for Assessment of Oxidative Stress in Cystic Fibrosis. Lung. août 2015;193(4):597-604.

4. Antus B, Harnasi G, Drozdovszky O, Barta I. Monitoring oxidative stress during chronic obstructive pulmonary disease exacerbations using malondialdehyde: MDA and COPD exacerbations. Respirology. janv 2014;19(1):74-9.

5. Antczak A, Nowak D, Shariati B, Król M, Piasecka G, Kurmanowska Z. Increased hydrogen peroxide and thiobarbituric acid-reactive products in expired breath condensate of asthmatic patients. European Respiratory Journal. 1 juin 1997;10(6):1235-41.

6. Araneda O, García C, Lagos N, Quiroga G, Cajigal J, Salazar M, et al. Lung oxidative stress as related to exercise and altitude. Lipid peroxidation evidence in exhaled breath condensate: a possible predictor of acute mountain sickness. Eur J Appl Physiol. déc 2005;95(5-6):383-90.

7. Araneda O, Guevara A, Contreras C, Lagos N, Berral F. Exhaled Breath Condensate Analysis after Long Distance Races. Int J Sports Med. 12 juill 2012;33(12):955-61.

8. Araneda OF, Urbina-Stagno R, Tuesta M, Haichelis D, Alvear M, Salazar MP, et al. Increase of pro-oxidants with no evidence of lipid peroxidation in exhaled breath condensate after a 10-km race in non-athletes. J Physiol Biochem. mars 2014;70(1):107-15.

9. Barregard L, Sallsten G, Andersson L, Almstrand AC, Gustafson P, Andersson M, et al. Experimental exposure to wood smoke: effects on airway inflammation and oxidative stress. Occupational and Environmental Medicine. 1 mai 2008;65(5):319-24.

10. Bartoli ML, Novelli F, Costa F, Malagrinò L, Melosini L, Bacci E, et al. Malondialdehyde in Exhaled Breath Condensate as a Marker of Oxidative Stress in Different Pulmonary Diseases. Mediators of Inflammation. 2011;2011:1-7.

11. Brand P, Bischof K, Siry L, Bertram J, Schettgen T, Reisgen U, et al. Exposure of healthy subjects with emissions from a gas metal arc welding process: part 3—biological effect markers and lung function. Int Arch Occup Environ Health. janv 2013;86(1):39-45.

12. Caglieri A, Goldoni M, Acampa O, Andreoli R, Vettori MV, Corradi M, et al. The Effect of Inhaled Chromium on Different Exhaled Breath Condensate Biomarkers among Chrome-Plating Workers. Environmental Health Perspectives. avr 2006;114(4):542-6.

13. Casimirri E, Stendardo M, Bonci M, Andreoli R, Bottazzi B, Leone R, et al. Biomarkers of oxidative-stress and inflammation in exhaled breath condensate from hospital cleaners. Biomarkers. 17 févr 2016;21(2):115-22.

14. Corradi M, Rubinstein I, Andreoli R, Manini P, Caglieri A, Poli D, et al. Aldehydes in Exhaled Breath Condensate of Patients with Chronic Obstructive Pulmonary Disease. Am J Respir Crit Care Med. 15 mai 2003;167(10):1380-6.

15. Corradi M, Alinovi R, Goldoni M, Vettori MV, Folesani G, Mozzoni P, et al. Biomarkers of oxidative stress after controlled human exposure to ozone. Toxicology Letters. août 2002;134(1-3):219-25.

16. Corradi M. Comparison between exhaled and sputum oxidative stress biomarkers in chronic airway inflammation. European Respiratory Journal. 1 déc 2004;24(6):1011-7.

17. Doruk S, Ozyurt H, Inonu H, Erkorkmaz U, Saylan O, Seyfikli Z. Oxidative status in the lungs associated with tobacco smoke exposure. Clinical Chemistry and Laboratory Medicine (CCLM) [Internet]. 1 janv 2011 [cité 30 mars 2022];49(12). Disponible sur: https://www.degruyter.com/document/doi/10.1515/CCLM.2011.698/html

18. Goldoni M, Catalani S, De Palma G, Manini P, Acampa O, Corradi M, et al. Exhaled Breath Condensate as a Suitable Matrix to Assess Lung Dose and Effects in Workers Exposed to Cobalt and Tungsten. Environmental Health Perspectives. sept 2004;112(13):1293-8.

19. Goldoni M, Corradi M, Mozzoni P, Folesani G, Alinovi R, Pinelli S, et al. Concentration of exhaled breath condensate biomarkers after fractionated collection based on exhaled CO_2 signal. J Breath Res. 27 févr 2013;7(1):017101.

20. Goldoni M, Caglieri A, Andreoli R, Poli D, Manini P, Vettori MV, et al. Influence of condensation temperature on selected exhaled breath parameters. BMC Pulm Med. déc 2005;5(1):10.

21. Gong J, Zhu T, Kipen H, Wang G, Hu M, Ohman-Strickland P, et al. Malondialdehyde in exhaled breath condensate and urine as a biomarker of air pollution induced oxidative stress. J Expo Sci Environ Epidemiol. mai 2013;23(3):322-7.

22. Graczyk H, Lewinski N, Zhao J, Sauvain JJ, Suarez G, Wild P, et al. Increase in oxidative stress levels following welding fume inhalation: a controlled human exposure study. Part Fibre Toxicol. déc 2015;13(1):31.

23. Gube M, Ebel J, Brand P, Göen T, Holzinger K, Reisgen U, et al. Biological effect markers in exhaled breath condensate and biomonitoring in welders: impact of smoking and protection equipment. Int Arch Occup Environ Health. oct 2010;83(7):803-11.

24. Inonu H, Doruk S, Sahin S, Erkorkmaz U, Celik D, Celikel S, et al. Oxidative Stress Levels in Exhaled Breath Condensate Associated With COPD and Smoking. Respiratory Care. 1 mars 2012;57(3):413-9.

25. Lärstad M, Ljungkvist G, Olin AC, Torén K. Determination of malondialdehyde in breath condensate by high-performance liquid chromatography with fluorescence detection. Journal of Chromatography B. janv 2002;766(1):107-14.

26. Laumbach RJ, Kipen HM, Ko S, Kelly-McNeil K, Cepeda C, Pettit A, et al. A controlled trial of acute effects of human exposure to traffic particles on pulmonary oxidative stress and heart rate variability. Part Fibre Toxicol. déc 2014;11(1):45.

27. Lee JS, Choi YC, Shin JH, Lee JH, Lee Y, Park SY, et al. Health surveillance study of workers who manufacture multi-walled carbon nanotubes. Nanotoxicology. 18 août 2015;9(6):802-11.

28. Majewska E, Kasielski M, Luczynski R, Bartosz G, Bialasiewicz P, Nowak D. Elevated exhalation of hydrogen peroxide and thiobarbituric acid reactive substances in patients with community acquired pneumonia. Respiratory Medicine. juill 2004;98(7):669-76.

29. Nowak D, Kałucka S, Białasiewicz P, Król M. Exhalation of H2O2 and thiobarbituric acid reactive substances (TBARs) by healthy subjects. Free Radical Biology and Medicine. janv 2001;30(2):178-86.

30. Pelclova D, Zdimal V, Schwarz J, Dvorackova S, Komarc M, Ondracek J, et al. Markers of Oxidative Stress in the Exhaled Breath Condensate of Workers Handling Nanocomposites. Nanomaterials. 10 août 2018;8(8):611.

31. Pelclová D, Fenclová Z, Vlčková Š, Klusáčková P, Lebedová J, Syslová K, et al. Occupational asthma follow-up — which markers are elevated in exhaled breath condensate and plasma? International Journal of Occupational Medicine and Environmental Health [Internet]. 1 janv 2014 [cité 30 mars 2022];27(2). Disponible sur: http://ijomeh.eu/Occupational-asthma-follow-up-Which-markers-are-elevated-in-exhaled-breath-condensate-and-plasma-,2044,0,2.html

32. Pelclova D, Zdimal V, Kacer P, Fenclova Z, Vlckova S, Syslova K, et al. Oxidative stress markers are elevated in exhaled breath condensate of workers exposed to nanoparticles during iron oxide pigment production. J Breath Res. 1 févr 2016;10(1):016004.

33. Riccelli MG, Goldoni M, Andreoli R, Mozzoni P, Pinelli S, Alinovi R, et al. Biomarkers of exposure to stainless steel tungsten inert gas welding fumes and the effect of exposure on exhaled breath condensate. Toxicology Letters. août 2018;292:108-14.

34. Rundell KW, Slee JB, Caviston R, Hollenbach AM. Decreased Lung Function After Inhalation of Ultrafine and Fine Particulate Matter During Exercise is Related to Decreased Total Nitrate in Exhaled Breath Condensate. Inhalation Toxicology. janv 2008;20(1):1-9.

35. Sakhvidi MJZ, Ardekani JB, Firoozichahak A, Zavarreza J, Hajaghazade M, Mostaghaci M, et al. Archive About the Journal Instructions for Authors Instructions for Reviewers Editorial Office Editorial Board Contact Reviewers 2014 2013 < PREVIOUS NEXT > ORIGINAL PAPER CC BY-NC 3.0 Polska Exhaled breath malondialdehyde, spirometric results and dust exposure assessment in ceramics production workers. Int J Occup Med Environ Health. 28 févr 2015;81-9.

36. Sauvain JJ, Hohl MSS, Wild P, Pralong JA, Riediker M. Exhaled Breath Condensate as a Matrix for Combustion-Based Nanoparticle Exposure and Health Effect Evaluation. Journal of Aerosol Medicine and Pulmonary Drug Delivery. déc 2014;27(6):449-58.

37. Stockfelt L, Sallsten G, Olin AC, Almerud P, Samuelsson L, Johannesson S, et al. Effects on airways of short-term exposure to two kinds of wood smoke in a chamber study of healthy humans. Inhalation Toxicology. janv 2012;24(1):47-59.

38. Syslová K, Kačer P, Kuzma M, Najmanová V, Fenclová Z, Vlčková Š, et al. Rapid and easy method for monitoring oxidative stress markers in body fluids of patients with asbestos or silica-induced lung diseases. Journal of Chromatography B. août 2009;877(24):2477-86.

39. Szkudlarek U, Maria L, Kasielski M, Kaucka S, Nowak D. Exhaled hydrogen peroxide correlates with the release of reactive oxygen species by blood phagocytes in healthy subjects. Respiratory Medicine. juin 2003;97(6):718-25.

40. Tanger SRO, éditeur. 10th Anniversary International Conference on Nanomaterials - Research and Application (NANOCON 2018): Brno, Czech Republic, 17-19 October 2018. Red Hook, NY: Curran Associates; 2019. 741 p.
